# Sertraline-induced rectal bleeding and anal pain (a rare case presentation)

**DOI:** 10.22088/cjim.13.1.136

**Published:** 2022

**Authors:** Pezhman Hadinezhad, Seyed Hamzeh Hosseini

**Affiliations:** 1 Department of Psychiatry, Psychiatry Research and Behavioral Sciences Institute, Mazandaran University of Medical Sciences, Sari, Iran

**Keywords:** Sertraline, Bleeding, Case reports

## Abstract

**Background:**

Selective serotonin reuptake inhibitors (SSRIs) have proven more problematic in terms of some side effects than the original clinical trials suggested. Sertraline may displace warfarin from plasma proteins and may increase the prothrombin time. The aim of this study was to report a rare case of the sertraline- induced severe anal pain and rectal bleeding without concurrent of taking any other drugs including non-steroidal anti-inflammatory drugs (NSAIDs).

**Case Presentation:**

Here we report a case of a 31 -year old married man who referred to a psychiatrist with depressive disorder and started to take sertraline up to 400 mg daily, thereafter the patient reported severe anal pain and bleeding. Other etiologies of this side effect were evaluated with Naranjo evaluation scale and rolled out. The patient did not report any anal pain or bleeding after eight months of stopping sertraline.

**Conclusion:**

Reported from sertraline, the psychiatrists must be more cautious when prescribing sertraline and monitor the patient properly for a long time to ensure these rare adverse effects and complications do not happen.

Although all selective serotonin reuptake inhibitors (SSRIs) are equally effective, there are meaningful differences in their pharmacodynamics and pharmacokinetics and side effects that might affect clinical responses among individual patients (1). The SSRIs have proven more problematic in terms of some side effects than the original clinical trials suggested. Quality of life is associated with adverse effects such as nausea, sexual dysfunction, and weight gain sometimes mitigate the therapeutic benefits of the SSRIs. Sertraline may displace warfarin from plasma proteins and may increase the prothrombin time (2). Moreover the SSRIs can cause functional impairment of platelet aggregation but not a reduction in platelet number. Easy bruising and excessive or prolonged bleeding manifest this pharmacological effect (3). Studies suggest that concurrent use of SSRIs and non-steroidal anti-inflammatory drugs (NSAIDs) is associated with a significantly increased risk of gastric bleeding (1). There are some reports throughout the world as regard to rare complications of sertraline including rhabdomyolysis, periorbital purpura, hepatotoxicity, acne, hypoglycemia, and hyponatremia (4-10). But there have been no reports globally including sertraline induced rectal bleeding and severe anal pain yet. The aim of this study was to report a rare case of the sertraline induced severe anal pain and rectal bleeding without concurrent of taking any other drugs including NSAIDs.

## Case presentation

Here we report a case of a 31-year-old married man who referred to a psychiatrist with depressed mood, anhedonia, loss of concentration, insomnia and decreased appetite. With diagnosis of major depressive disorder, the psychiatrist started treatment with prescribing sertraline 50 mg daily. The patient also had no history of gastrointestinal (GI) problems or any medical complications, nor family history of gastrointestinal bleeding. He only had a history of taking acetaminophen codeine when he had occasionally headaches, and he did not report any adverse effects. 

He had not history of using NSAIDs or other drugs or substances either. Due to less effectiveness of treatment for depression, sertraline dose increased up to 400 mg daily within two months. Although after that depression symptoms subsided, the patient reported severe anal pain and bleeding. Hence he was referred to psychiatrist again and stopped taking sertraline. His other laboratory findings such as endoscopy and colonoscopy and also physical examination were normal and other etiologies of this side effect were evaluated with Naranjo evaluation scale and rolled out. The patient did not report any anal pain or bleeding after eight months of stopping sertraline.

## Discussion

Although multiple studies showed that SSRIs can increase the bleeding time or platelets dysfunction, the interesting note about this case was that he had neither physical problem, nor history of peptic ulcer or gastrointestinal bleeding. In addition, there is no study that reports severe anal pain and bleeding due to sertraline treatment. Also this report is unique in the sense that the adverse effect of SSRIs, especially bleeding, occurred when the patient use concurrent drugs such as aspirin and other NSAIDs. But in this case report, the patient had not been using any other drugs except acetaminophen codeine occasionally. Whether or not concurrency of acetaminophen codeine and sertraline use could have been a question and needs more study. Among the global reports regarding sertraline was a report of microscopic colitis in case of a 63- year- old Caucasian man without any previous gastrointestinal problems. The patient had been using sertraline 100 mg daily with 0.5 mg alprazolam daily for treatment of mixed anxiety depressive disorder (11). 

The main complaint of this case was diarrhea while in the recent case, he had complaints about severe anal pain with bleeding, and the side effects happened in a younger age, and he had used a higher dose of sertraline. However, both patients had no GI problems or medical complications. Other reports aside from gastrointestinal system such as diplopia, hemi chorea, dysgraphia, acute eosinophilic pneumonia, galactorrhea, and akathisia have been reported globally as rare sertraline complications (12-17). 

But there have no similar reports about the recent complication. Therefore, although in authentic medical sources, the SSRIs are known as safe and low-risk medicines, considering multiple rare complications reported from sertraline, the psychiatrists must be more cautious when prescribing sertraline and monitor the patients properly for long time to ensure these rare adverse effects and complications do not happen.

## Conflict of interest:

Authors declare that there is no conflict of interest

## References

[1] Sadock BJ, Sadock Virginia A, Ruiz P (2015). Kaplan and Sadocks synopsis of psychiatry.

[2] Apseloff G, Wilner KD, Gerber N, Tremaine LM (1997). Effect of sertraline on protein binding of warfarin. Clin Pharmacokinet.

[3] Serebruany VL, Gurbel PA, O'Connor CM (2001). Platelet inhibition by sertraline and N-desmethylsertraline: a possible missing link between depression, coronary events, and mortality benefits of selective serotonin reuptake inhibitors. Pharmacol Res.

[4] Holmberg PJ, Arteaga G, Schiltz BM, Homme J (2018). Sertraline-Induced rhabdomyolysis, trismus, and cardiac arrest in a child. Pediatrics.

[5] Kayhan F, Eken ZE, Uguz F (2015). Sertraline-induced periorbital purpura: a case report. Australas Psychiatry.

[6] Collados Arroyo V, Plaza Aniorte J, Hallal H, Perez-Cuadrado E (2008). Sertraline-induced hepatotoxicity]. Farm Hosp.

[7] Sinha S, Udupa S, Bhandary RP, Praharaj SK, Munoli RN (2014). Sertraline-induced acneiform eruption. J Neuropsychiatry Clin Neurosci.

[8] Pollak PT, Mukherjee SD, Fraser AD (2001). Sertraline-induced hypoglycemia. Ann Pharmacother.

[9] Papelbaum M, Aguiar MC (2006). Sertraline-induced hyponatraemia. Braz J Psychiatry.

[10] Maramattom BV, Thomas J, Kachhare N (2018). Sertraline-induced reversible myopathy with rhabdomyolysis and trismus. Neurology India.

[11] Menon R, Ng C (2015). Sertraline-induced microscopic colitis. Psychosomatics.

[12] Alao A, Lewkowicz C (2015). Seeing double: sertraline and diplopia: a case report. Int J Psychiat Med.

[13] Gatto EM, Aldinio V, Parisi V (2017). Sertraline-induced hemichorea. Tremor Other Hyperkinet Mov (N Y).

[14] Gentili M, Marinaccio PM, Galimberti C (2016). A case of dysgraphia induced by sertraline and a review of official spontaneous adverse reaction databases. J Clin Pharm Ther.

[15] Muftah M, Nassri R, Nassri A, Harford P (2018). Sertraline-induced acute eosinophilic pneumonia. Curr Drug Saf.

[16] Nebhinani N (2013). Sertraline-induced galactorrhea: case report and review of cases reported with other SSRIs. Gen Hosp Psychiatry.

[17] Nepal H, Black E, Bhattarai M (2016). Self-Harm in Sertraline-Induced Akathisia. Prim Care Companion CNS Disord.

